# Development and application of a new measure of engagement in out‐patient HIV care

**DOI:** 10.1111/hiv.12427

**Published:** 2016-08-18

**Authors:** AR Howarth, FM Burns, V Apea, S Jose, T Hill, VC Delpech, A Evans, CH Mercer, S Michie, S Morris, M Sachikonye, C Sabin, the REACH study, the UK Collaborative HIV Cohort (UK CHIC) study, Jonathan Ainsworth, Sris Allan, Jane Anderson, Abdel Babiker, David Chadwick, David Dunn, Martin Fisher, Brian Gazzard, Richard Gilson, Mark Gompels, Phillip Hay, Margaret Johnson, Stephen Kegg, Clifford Leen, Fabiola Martin, Mark Nelson, Chloe Orkin, Adrian Palfreeman, Andrew Phillips, Deenan Pillay, Frank Post, Jillian Pritchard, Achim Schwenk, Anjum Tariq, Roy Trevelion, John Walsh, Alicia Thornton, Susie Huntington, Adam Glabay, N. Perry, S. Tilbury, E. Youssef, D. Churchill, R. Everett, D. Asboe, S. Mandalia, H. Korat, C. Taylor, Z. Gleisner, F. Ibrahim, L. Campbell, N. Brima, I. Williams, M. Youle, F. Lampe, C. Smith, R. Tsintas, C. Chaloner, S. Hutchinson, N. Mackie, A. Winston, J. Weber, F. Ramzan, M. Carder, J. Lynch, J. Hand, C. Souza, J. Anderson, S. Munshi, S. Miller, C. Wood, C. Leen, A. Wilson, S. Morris, K. Memon, A. Lewszuk, E. Cope, J. Gibson, P. Main, S. Mitchell, M. Hunter, M. Dhillon, S. Russell‐Sharpe, A. Harte, S. Clay, A. Tariq, H. Spencer, R. Jones, S. Cumming, C. Atkinson

**Affiliations:** ^1^ Research Department of Infection and Population HealthUniversity College LondonLondonUK; ^2^ Barts Health NHS TrustLondonUK; ^3^ Public Health EnglandLondonUK; ^4^ Royal Free London NHS Foundation TrustLondonUK; ^5^ Centre for Behaviour ChangeUniversity College LondonLondonUK; ^6^ Department of Applied Health ResearchUniversity College LondonLondonUK; ^7^ UK Community Advisory BoardLondonUK

**Keywords:** cohort study, HIV, out‐patient care, patient engagement, retention measure

## Abstract

**Objectives:**

Commonly used measures of engagement in HIV care do not take into account that the frequency of attendance is related to changes in treatment and health status. This study developed a new measure of engagement in care (EIC) incorporating clinical factors.

**Methods:**

We conducted semi‐structured interviews with eight HIV physicians to identify factors associated with the timing of patients' next scheduled appointments. These factors informed the development of an algorithm to classify each month of follow‐up as “in care” (on or before the time of the next expected attendance) or “out of care” (after the time of the next expected attendance). The EIC algorithm was applied to data from the UK Collaborative HIV Cohort (UK CHIC) study, a large clinical cohort study.

**Results:**

The interviews indicated that time to next appointment varied depending on psychosocial and physical comorbidities, and clinical factors (time since diagnosis, AIDS diagnosis, treatment status, CD4 count and viral load). The resulting EIC algorithm was applied to 44 432 patients; 83.9% of the 3 021 224 person‐months were “in care”. Greater EIC was independently associated with older age, white ethnicity, HIV acquisition through sex between men, current use of antiretroviral therapy (ART), a higher nadir CD4 count, later calendar year and being seen at the clinic for the first time within the last year.

**Conclusions:**

This algorithm describing engagement in HIV care incorporates a time‐updated measure of patients' treatment and health status. It adds to the options available for measuring this key performance indicator.

## Introduction

The introduction of combination antiretroviral therapy (ART) has led to a dramatic reduction in HIV‐associated morbidity and mortality 1. While the life expectancy for successfully treated people living with HIV in the UK is now similar to that of the general population 2, patients who do not attend all their HIV clinic appointments remain at higher risk of long‐term mortality 3. ART is also recognized as an effective means of reducing HIV transmission 4 and yet the individual and public health benefits of HIV treatment can only be achieved if people living with HIV are aware that they are HIV positive and have sustained engagement with care.

Engagement in out‐patient HIV care is therefore a key measure of quality performance for HIV service providers 5 and a number of measures have been proposed. The number or proportion of missed appointments has been used where these data are available 3, 6. In the absence of appointment data, measures often rely on laboratory data as surrogate markers of attendance. This can be used to measure visit constancy 6, which assesses the proportion of time intervals in which patients attend for care on at least one occasion: a recent Danish study, for example, used the proportion of person‐years where patients had at least one contact with the HIV care system 7. Another study defined suboptimal care as years when fewer than two CD4 count or viral load measurements occurred per calendar year 8. The Health Resources and Services Administration HIV/AIDS Bureau (HRSA HAB) measure has been used to examine the proportion of years where patients have at least two out‐patient visits separated by 90 days 9, 10. A comparison of a measure based on gaps between visits of more than 6 months with a 91‐day measure of constancy and the HRSA HAB measure found moderately strong correlation between measures 11.

There is no gold standard measure of engagement in HIV out‐patient care. While each of the above measures has its own strengths and weaknesses 6, none of them takes into account that the frequency of attendance is related to changes in treatment and health status and may also be affected by external forces or changes in clinic policy. In the UK, for example, British HIV Association (BHIVA) guidelines indicate that patients should be seen within 2–4 weeks of starting ART and every 3–6 months for routine monitoring on ART if they are considered “stable” and have good adherence and an undetectable viral load 12. More recently, however, clinics have switched to a policy of less frequent monitoring in this subgroup in accordance with best practice and in order to manage an increasing clinic load in the setting of reduced resources for HIV health care. A recent study suggests that annual CD4 monitoring may be appropriate for virally supressed patients with a baseline CD4 count of ≥ 250 cells/μL 13.

The Retention and Engagement Across Care Services for HIV (REACH) study set out to better understand HIV out‐patient attendance in order to develop cost‐effective interventions to optimize engagement in care (EIC). Taking into account that the frequency of monitoring is dependent on treatment and health status, and as gaps between clinic visits may vary quite considerably within the current guidelines 12, we conducted interviews with physicians about the factors that influence the timing of a patient's next scheduled appointment. The information was used to inform the development of an algorithm that can be used to provide a measure of EIC that is sensitive to changes in an individual's status over time. The aim of this present study was to describe the initial development of this algorithm and its application to a large clinical cohort in the UK.

## Methods

### Algorithm development

Exploratory, semi‐structured, face‐to‐face interviews were conducted with eight HIV physicians with a range of clinical experience selected from five HIV out‐patient clinics in inner and outer London where the prevalence of HIV is high 14. The physicians were purposively selected to ensure representation from each of the five clinics which had previously agreed to participate in the REACH study. They were of various sizes and covered different patient populations in north, east and central London. All clinics were based in specialist services for sexual health and HIV and attended by general HIV out‐patients.

The interviews, which were conducted by AH, took 25–30 min and were recorded verbatim. Physicians were asked to describe the factors that prompted the timing of the next scheduled appointment for the last ten patients that they had seen: specifically, they were asked when they had asked to see the patient again (number of weeks/months) and why. Interviews took place from November 2013 to February 2014 and the findings therefore reflect guidelines in place at the time 12. Physicians were asked not to provide any information that would identify patients.

We conducted a content analysis 15 of these qualitative data. For each patient, we noted the time to the next scheduled appointment and the key reason for the timing of this appointment given by their physician. We then identified factors under which to code the key reasons. The data were entered into spss statistics 22 (IBM Corporation, Armonk, New York, USA) to produce a cross‐tabulation of time to appointment by factor. The findings from this analysis informed the development of an algorithm to measure EIC that was refined iteratively to the precision required for programming.

### Application of the algorithm to a clinical data set

The algorithm was applied to data from the UK Collaborative HIV Cohort (UK CHIC) study and analyses were performed using sas 9.3 (SAS Institute, Cary, NC). UK CHIC collates routine data on HIV‐positive people, aged 16 years or older, who have attended some of the largest HIV clinics in the UK since 1 January 1996. For this analysis, we included all patients who attended a participating UK CHIC clinic on two or more occasions between 1 January 2000 and 31 December 2012. In the absence of complete data on clinic attendances, CD4 counts, viral loads, haemoglobin measurements and ART start or switch dates were used as markers of clinic attendance. Follow‐up for each person was considered to continue until the last recorded laboratory marker or clinic visit prior to (or on) 31 December 2012.

**Figure 1:**
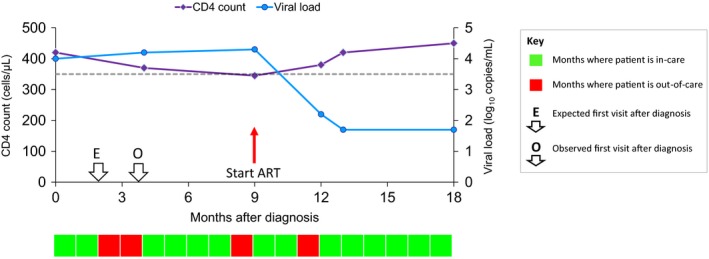
Measure of engagement in HIV care applied to an individual case. In this example, the patient was diagnosed with a CD4 count of 420 cells/μL and a viral load of 4.0 log_10_ copies/mL. As she was diagnosed during this care episode, we expect to see her again within 2 months (E). However, she did not re‐attend until 4 months after diagnosis (O). Thus, months 1 and 2 are defined as being in care (light shading) and months 3 and 4 as being out of care (dark shading). By the time of her next care episode, her CD4 count had fallen to 370 cells/μL so we expect to see her again within 4 months but she actually re‐attended after 5 months; thus, months 5–8 are in care and month 9 is out of care. At the next care episode, she started antiretroviral therapy (ART) so we expect to see her again within 2 months. As she did not re‐attend until 3 months later, months 10 and 11 are defined as in care and month 12 is out of care. She then re‐attended after 1 month, by which time her CD4 count was back up to 420 cells/μL and her viral load was undetectable ‐ which means that we would not expect to see her for another 6 months (with follow‐up ending at 5 months in this example).

Individuals may often have repeat laboratory tests performed within a short time interval to confirm unexpected findings, and this may result in clusters of measurements around a single “index” date. For our analysis, we did not want to consider each of the measurements within a cluster as independent visits, as only the index visit would have been scheduled at the previous visit. Thus, we grouped attendances into “care episodes”, defined as months (period of 30.4 days since entry into the study) where at least one visit occurred. For each care episode, we then established the lowest CD4 count measured in that month (and the change from the previous value), the highest HIV viral load (and the status of this measurement relative to other consecutive values) and the patient's treatment status, and used this information to establish a likely date of next scheduled visit using our algorithm (see Results and Fig. 1 for an example of the application of the algorithm). The date of the next observed care episode determined whether the patient had attended before or after the expected date, and each patient‐month was then classified as being in care (where it occurred on or before the time of the next expected care episode) or out of care (where it occurred after the time of the next expected care episode) accordingly.

### Statistical methods

The proportion of months where patients were engaged in HIV care was calculated overall and for patient subgroups defined by gender, age group (< 25, 25–45 and > 45 years), ethnic group (white, black African, other and unknown), mode of HIV acquisition (sex between men, sex between men and women, injecting drug use and other/unknown), whether currently on ART, nadir and current CD4 counts (both classified as < 200, 200–349 and ≥ 350 cells/μL), participating clinic, calendar year (2000–2003, 2004–2007 and 2008–2012) and time since entry in the study (< 1, 1–5, 5–10 and > 10 years). Note that we did not consider follow‐up after the patient's last reported care date, and therefore this algorithm focuses on intermittent periods of disengagement rather than ultimate loss to follow‐up after the person's last clinic visit. Each patient‐month was then treated as a separate entry in a multivariable logistic regression model with the aim of identifying demographic and clinical factors associated with that month being “in care”. These models were fitted using proc genmod in sas, with generalized estimating equations being used to take account of the repeated entries within each individual patient.

## Results

### Qualitative interviews with physicians

A total of 73 patients were discussed in the physician interviews. One patient was not included in the analysis because their next appointment was dependent on awaited test results. The time of the next scheduled appointment was missing for another patient and not available for a further five patients who had not attended their last scheduled appointment at the time of the physician interviews.

The time of the next scheduled appointment in the remaining 66 patients ranged from 1 week to 6 months, with a median of 3 months. While physicians were acting within the current guidelines, they described the reasons for this variation in the timing of the next scheduled appointment. Five factors were identified from the content analysis of the interview data as instrumental. These factors were summarized as “routine” where patients were stable and required routine follow‐up, “virological” where the next appointment was based on change in viral load (uncontrolled or virological breakthrough), “treatment” where the next appointment was related to starting ART or changing an existing ART regimen, “psychosocial” where mental health or psychosocial issues were identified as instrumental, and “physical comorbidities” where a range of physical comorbidities were given as the key reason for the timing of the next appointment.

One‐third of patients were described as stable and given routine follow‐up appointments 4–6 months after their last visit. Routine follow‐up for one pregnant woman was arranged for 3 months' time. Physicians talked about extending routine visits to every 6 months when patients were well and stable, both on treatment and in their psychosocial circumstances. Changes in viral load brought the next scheduled appointment forward to 1–2 months after the last. Patients who were starting or changing treatment were given a next appointment date between 2 and 12 weeks later, depending on the treatment start date and virological response or when treatment was planned to start. Follow‐up appointments of between 1 week and 4 months later were arranged depending on a range of psychosocial issues (from specific concerns about mental health to more general needs for social support) and comorbidities: both of which required earlier follow‐up when patients were often otherwise stable on treatment.

### Algorithm development

We used the data from the physician interviews on the timing of next appointments as the basis for developing the EIC algorithm. Although psychosocial wellbeing and comorbidities were key factors in determining the expected time between patient visits to the HIV clinic, data on these variables are not generally captured electronically and are not often available in routinely collected cohort data. Thus, we used clinical data (HIV diagnosis, AIDS diagnosis, treatment start dates, CD4 count and viral load) only to determine the patient's treatment and health status. This was used to estimate the expected time to the next scheduled care episode, in accordance with the data collected in the physician interviews (Table 1). The EIC algorithm was then refined for programming.

**Table 1:** Conditions associated with the expected time of the next scheduled care episode

Conditions at time of initial care episode^a^	Next care episode expected within
Within 1 month of HIV diagnosis	2 months
AIDS diagnosis	2 months
Started ART	2 months
Started new combination	2 months
Not on ART	
CD4 count ≤ 350 cells/μL any drop in CD4 count	2 months
CD4 count ≤ 350 cells/μL; no drop in CD4 count	4 months
CD4 count 351–499 cells/μL	4 months
CD4 count ≥ 500 cells/μL; CD4 count drop ≥ 100 cells/μL	4 months
CD4 count ≥ 500 cells/μL; CD4 count drop < 100 cells/μL; viral load ≥ 100 000 copies/mL	4 months
CD4 count ≥ 500 cells/μL; CD4 count drop < 100 cells/μL; viral load < 100 000 copies/mL	6 months
Already started ART	
Viral load > 200 copies/mL	2 months
Viral load 51–200 copies/mL; does not appear to be blip^b^	2 months
Viral load 51–200 copies/mL; appears to be blip	4 months
Viral load ≤ 50 copies/mL; CD4 count ≤ 200 cells/μL	4 months
Viral load ≤ 50 copies/mL CD4 count > 200 cells/μL	6 months

ART, antiretroviral therapy.

a If more than one condition applies at the time of the care episode, the next care episode is expected within the least number of months associated with those conditions.

b Blips are defined as having a viral load of between 50 and 200 HIV‐1 RNA copies/mL following a previous viral load of < 50 copies/mL.

According to the EIC algorithm, the shortest expected gap between care episodes was 2 months. This is to allow for the fact that clinic visits might occur at any point during the month or care episode into which they are grouped. If the patient was within 1 month of diagnosis, had an AIDS diagnosis, or started ART or changed ART at the initial care episode, the next care episode was expected within 2 months. If the patient was not on ART at the initial care episode, the next care episode was expected within 2–6 months, depending mainly on CD4 count. If the patient had started ART, it was expected within 2–6 months, depending on viral load. We used 6 months as the maximum time between visits, as described in the physician interviews. If more than one condition applied at the time of the initial care episode, the next care episode was expected within the least number of months associated with those conditions.

Fig. 1 shows an example of how the EIC algorithm is applied to an individual case. In this example, the patient was out of care for 4 of her 18 months of follow‐up and was therefore in care for 14/18 = 77.8% of months.

### Associations between engagement in care and factors identified in UK CHIC

A total of 44 432 patients from UK CHIC (2000–2012) were included in the following analysis. Women represented 27.8% of the sample. Half were white (53.3%), one‐third were black African (28.9%), 8.7% were of other ethnicity and 9.2% had unknown ethnicity. Around half had acquired HIV through sex between men (50.5%), with 39.1% acquiring HIV through sex between men and women, 3.0% through injecting drug use and the remaining 7.4% through other or unknown routes. Their median age at entry into the study was 36 years [interquartile range (IQR) 30–42 years] and the median date of follow‐up start was December 2004 (range January 2000 to October 2012). The median CD4 count at start of follow‐up was 355 (IQR 214–520) cells/μL; patients were followed for a median of 61 (range 2–156) months with a total follow‐up of 3 021 224 patient‐months.

Overall, patients were engaged in care for 83.9% of the total follow‐up of patient‐months. Table 2 shows the proportion of months that were engaged in care stratified by the various demographic and clinical factors, as well as the results of univariable and multivariable regression models. In univariable analyses, EIC was higher in men, in those aged > 45 years, in those of white ethnicity, in those who acquired HIV through sex between men, in those with higher nadir and current CD4 counts, in later calendar years and in those who had only recently (within the last year) been first seen at the clinic. After adjustment for other factors shown in Table 2 and for clinic, most of these associations were unchanged with three main exceptions. Firstly, there was no strong association between gender and EIC. Secondly, while current use of ART did not appear to be associated with EIC in unadjusted analyses, after adjustment it became apparent that those currently on ART had higher levels of engagement. Finally, including adjustment for the nadir CD4 count showed that current CD4 count did not provide any independent association with EIC.

**Table 2:** Unadjusted and adjusted associations with retention in care in any particular month

Factor				Unadjusted			Adjusted^a^		
		Person‐months	% retention in care	OR	(95% CI)	*P*‐value	OR	(95% CI)	*P*‐value
Gender	Male	2 235 135	85.1	1.36	(1.31, 1.40)	0.0001	1.10	(0.98, 1.23)	0.11
	Female	786 089	80.7	1	–	–	1	–	–
Age group	< 25 years	83 116	77.1	0.63	(0.42, 0.93)	0.02	0.67	(0.42, 1.06)	0.09
	25–45 years	1 960 061	82.5	0.66	(0.53, 0.81)	0.0001	0.74	(0.59, 0.93)	0.008
	> 45 years	978 023	87.4	1	–	–	1	–	–
Ethnic group	White	1 760 442	85.5	1	–	–	1	–	–
	Black African	802 477	81.2	0.74	(0.71, 0.76)	0.0001	0.96	(0.83, 1.11)	0.55
	Other	239 190	81.8	0.77	(0.73, 0.81)	0.0001	0.79	(0.68, 0.92)	0.002
	Unknown	219 115	83.6	0.87	(0.82, 0.92)	0.0001	0.87	(0.73, 1.03)	0.11
Route of acquisition	MSM	1 687 095	86.2	1	–	–	1	–	–
	IDU	94 014	76.3	0.52	(0.48, 0.56)	0.0001	0.56	(0.44, 0.71)	0.0001
	Heterosexual	1 127 473	81.4	0.70	(0.68, 0.73)	0.0001	0.84	(0.73, 0.98)	0.02
	Other/unknown	112 642	82.2	0.77	(0.71, 0.83)	0.0001	0.80	(0.67, 0.95)	0.01
Currently on ART	No	616 201	74.6	1	–	–	1	–	–
	Yes	2 405 023	86.3	0.95	(0.79, 1.15)	0.62	1.44	(1.15, 1.81)	0.002
Nadir CD4 count	< 200 cells/μL	1 528 352	87.8	0.51	(0.40, 0.65)	0.0001	0.55	(0.41, 0.74)	0.0001
	200–349 cells/μL	821 951	84.3	0.33	(0.25, 0.45)	0.0001	0.37	(0.28, 0.50)	0.0001
	≥ 350 cells/μL	571 445	76.7	1	–	–	1	–	–
Current CD4 count	< 200 cells/μL	256 512	80.8	0.68	(0.53, 0.87)	0.002	0.82	(0.64, 1.05)	0.12
	200–349 cells/μL	587 648	82.7	0.81	(0.69, 0.94)	0.006	0.93	(0.81, 1.08)	0.34
	≥ 350 cells/μL	2 077 588	85.7	1	–	–	1	–	–
Calendar year	2000–2003	553 178	82.5	0.59	(0.50, 0.70)	0.0001	0.61	(0.51, 0.72)	0.0001
	2004–2007	1 500 392	85.2	1	–	–	1	–	–
	2008–2012	967 654	82.8	1.68	(1.45, 1.94)	0.0001	1.71	(1.47, 1.98)	0.0001
Time since	< 1 year	351 190	87.4	1.80	(1.34, 2.40)	0.0001	1.53	(1.09, 2.15)	0.01
Entry in UK	1–5 years	1 137 979	82.5	1.19	(0.89, 1.59)	0.24	1.24	(0.89, 1.71)	0.20
CHIC	5–10 years	1 020 656	83.4	1.22	(0.92, 1.61)	0.16	1.13	(0.85, 1.51)	0.41
	> 10 years	511 399	85.8	1	–	–	1	–	–

ART, antiretroviral therapy; CI, confidence interval; IDU, injecting drug use; MSM, men who have sex with men; OR, odds ratio; UK CHIC, UK Collaborative HIV Cohort.

a Adjusted for other variables shown in the table and for clinic.

## Discussion

We have developed an algorithm which provides a flexible new approach to measuring engagement in out‐patient HIV care. It is, to our knowledge, the first measure that adapts to the changing treatment and health status of the patient, reflecting the reality described to us by physicians and giving it strong face validity 6. The EIC algorithm can also be easily modified at a clinic level and/or over time to reflect changes in service delivery or treatment criteria, or when comparing EIC across different sites with different monitoring frequencies.

The algorithm provides a dichotomous measure for each month of follow‐up as to whether the patient is in care or out of care. Poor EIC during the first year after diagnosis is associated with a higher rate of mortality 3 and the algorithm may be usefully applied to this period, as shown in our illustrative example, in addition to other key short periods of time, such as the first year after giving birth. It can be used in longitudinal analysis of patterns of engagement over extended periods of time 16 and to examine associations between predictive variables and the proportion of months that patients are in care following diagnosis.

We found that patients were engaged in care for 83.9% of months over a follow‐up period of up to 12 years. Consistent with findings from an analysis of loss to follow‐up in the annual Survey of Prevalent HIV Infections Diagnosed in England, Wales and Northern Ireland 17, greater engagement in HIV care was more likely in men who have sex with men, and those who were less engaged were more likely to be women, of black ethnicity and younger. Studies using a range of measures of retention and engagement have also found that HIV patients are less likely to disengage from care if they are older 18, 19, 20, white 21, men who have sex with men 21, 22, and have started ART 17, 18. The consistency in these findings provides a measure of external validity for the algorithm.

The EIC algorithm was developed from interviews with physicians who indicated that the timing between appointments is dependent on a range of factors. The interviews clearly illustrate that there is no one‐size‐fits‐all when it comes to the timing of HIV clinic appointments. Patients stable on treatment were seen for routine care every 4 months which was extended to every 6 months, as appropriate for the individual under care, guided by the therapeutic relationship. We have used 6 months as the maximum time between routine visits in the algorithm, as described by physicians in accordance with the UK guidelines for routine monitoring 12. However, the EIC algorithm could be adapted to changing guidelines for monitoring HIV patients and to local clinic policies on how often to see patients. For example, new treatment guidelines in the UK recommend starting ART irrespective of CD4 count 23 and this should be incorporated into the EIC algorithm when it is applied to future cohort data.

The majority (90%) of people who are being seen for HIV care in the UK are on ART 14. This was also the case among patients discussed in our physician interviews and the data they provided on response to treatment and virological breakthrough informed the development of the EIC algorithm. Psychosocial issues and comorbidities also played a key role in the timing of patients' next scheduled appointments – although this finding may be limited by an over‐representation of more complex patients in our study, reflecting the clinical interests and patient cohorts of the physicians who took part in our interviews. Patients affected by these factors were scheduled to come back within a shorter period of time, from 1 week to 4 months. While such data are not currently collected for UK CHIC, they could be incorporated into an algorithm if they were available. For example, the new UK HIV and AIDS Reporting System (HARS) 24 includes a measure of patient complexity that could be incorporated into future iterations of the EIC algorithm. The EIC algorithm may therefore provide an under‐estimation of engagement in HIV care as it does not account for patients whom physicians may wish to see earlier for treatment of comorbidities and psychosocial issues associated with HIV.

In common with other analyses of EIC using HIV cohort data, we have used laboratory data and ART start dates as surrogate markers of clinic visits. It is possible, therefore, that we may have missed some visits where no laboratory test was performed. Further analyses of data from the group of clinics that are able to provide more detailed information on attendances will allow us to validate this approach. We grouped visits into care episodes to negate the effect of repeated laboratory measurements within short time intervals.

We censored the data at the last recorded visit and, while our measure does not therefore include any ongoing periods of loss to follow‐up, it would be possible to incorporate this into the algorithm. It should also be noted that any algorithm is clearly only an approximation to a far more complex clinical process and it is difficult in an observational cohort setting to incorporate other factors, such as social factors, that may lead to more regular scheduled visits. This is, however, likely to result in an under‐estimation of EIC rather than an over‐estimation. While our algorithm has these limitations, we have created a measure of engagement in HIV care that will benefit from the advantages of using these data 25, with the associated years of follow‐up, statistical power and representative patient populations. The EIC algorithm can be used to examine how patients engage in HIV care over time and identify variables associated with disengagement, with the aim of achieving the best possible health outcomes for all 1. In future work, we will consider the associations between EIC and longer term outcomes among individuals receiving ART.

EIC is a key quality performance measure for HIV service providers and it is important that it is captured in a way that reflects whether patients are attending as frequently as indicated by their clinical needs. We have presented a concept of how to measure engagement in HIV care by incorporating a time‐updated measure of patients' treatment and health status and a prototype of this measure that we have tested on HIV cohort data. The EIC algorithm adds to the options available for measuring engagement in HIV care, and assessing this key performance indicator.

## Appendix a The REACH study

REACH Study Steering Committee: Paul Clift, Paul Flowers, Jonathan Sterne, Ann Sullivan.

REACH Participating Centres: Barts Health NHS Trust (V. Apea); Chelsea and Westminster Hospital NHS Foundation Trust (A. Sullivan); Homerton University Hospital NHS Foundation Trust (I. Reeves); King's College Hospital NHS Foundation Trust (J. Fox); Mortimer Market Centre, University College London (A. Milinkovic); Royal Free London NHS Foundation Trust (F. Burns).

## Appendix b The UK CHIC study

UK CHIC Steering Committee: Jonathan Ainsworth, Sris Allan, Jane Anderson, Abdel Babiker, David Chadwick, Valerie Delpech, David Dunn, Martin Fisher, Brian Gazzard, Richard Gilson, Mark Gompels, Phillip Hay, Teresa Hill, Margaret Johnson, Sophie Jose, Stephen Kegg, Clifford Leen, Fabiola Martin, Mark Nelson, Chloe Orkin, Adrian Palfreeman, Andrew Phillips, Deenan Pillay, Frank Post, Jillian Pritchard, Caroline Sabin, Memory Sachikonye, Achim Schwenk, Anjum Tariq, Roy Trevelion and John Walsh.

UK CHIC Central Co‐ordination: University College London (Teresa Hill, Sophie Jose, Andrew Phillips, Caroline Sabin, Alicia Thornton and Susie Huntington); Medical Research Council Clinical Trials Unit at UCL (MRC CTU at UCL), London (David Dunn and Adam Glabay).

UK CHIC Participating Centres: Brighton and Sussex University Hospitals NHS Trust (M. Fisher, N. Perry, S. Tilbury, E. Youssef and D. Churchill); Chelsea and Westminster Hospital NHS Foundation Trust, London (B. Gazzard, M. Nelson, R. Everett, D. Asboe and S. Mandalia); King's College Hospital NHS Foundation Trust, London (F. Post, H. Korat, C. Taylor, Z. Gleisner, F. Ibrahim and L. Campbell); Mortimer Market Centre, University College London (R. Gilson, N. Brima and I. Williams); Royal Free NHS Foundation Trust/University College London (M. Johnson, M. Youle, F. Lampe, C. Smith, R. Tsintas, C. Chaloner, S. Hutchinson, C. Sabin, A. Phillips, T. Hill, S. Jose, A. Thornton and S. Huntington); Imperial College Healthcare NHS Trust, London (J. Walsh, N. Mackie, A. Winston, J. Weber, F. Ramzan and M. Carder); Barts and The London NHS Trust, London (C. Orkin, J. Lynch, J. Hand and C. de Souza); Homerton University Hospital NHS Trust, London (J. Anderson and S. Munshi); North Middlesex University Hospital NHS Trust, London (J. Ainsworth, A. Schwenk, S. Miller and C. Wood); The Lothian University Hospitals NHS Trust, Edinburgh (C. Leen, A. Wilson and S. Morris); North Bristol NHS Trust (M. Gompels and S. Allan); Leicester, University Hospitals of Leicester NHS Trust (A. Palfreeman, K. Memon and A. Lewszuk); Middlesbrough, South Tees Hospitals NHS Foundation Trust (D. Chadwick, E. Cope and J. Gibson); Woolwich, Lewisham and Greenwich NHS Trust (S. Kegg, P. Main, S. Mitchell and M. Hunter), St George's Healthcare NHS Trust (P. Hay and M. Dhillon); York Teaching Hospital NHS Foundation Trust (F. Martin and S. Russell‐Sharpe); Coventry, University Hospitals Coventry and Warwickshire NHS Trust (S. Allan, A. Harte and S. Clay); Wolverhampton, The Royal Wolverhampton Hospitals NHS Trust (A. Tariq, H. Spencer and R. Jones); Chertsey, Ashford and St Peter's Hospitals NHS Foundation Trust (J. Pritchard, S. Cumming and C. Atkinson); Public Health England, London (V. Delpech); UK Community Advisory Board (M. Sachikonye and R. Trevelion).
